# Relationship between Punitive Discipline and Child-to-Parent Violence: The Moderating Role of the Context and Implementation of Parenting Practices

**DOI:** 10.3390/ijerph19010182

**Published:** 2021-12-24

**Authors:** M. Carmen Cano-Lozano, Samuel P. León, Lourdes Contreras

**Affiliations:** 1 Department of Psychology, University of Jaén, 23071 Jaén, Spain; lmcontre@ujaen.es; 2 Department of Education, University of Jaén, 23071 Jaén, Spain; sparra@ujaen.es

**Keywords:** child-to-parent violence, punitive discipline, parental stress, parental ineffectiveness, parental impulsivity, parental warmth/support

## Abstract

This study examines the influence of punitive parental discipline on child-to-parent violence (CPV). The moderating roles of parental context (stress and parental ineffectiveness), mode of implementation of parental discipline (parental impulsivity or warmth/support) and the gender of the aggressor in the relationship between punitive discipline and CPV are examined. The study included 1543 university students between 18 and 25 years old (50.2% males, M_age_ = 19.9 years, SD = 1.9) who retrospectively described their experience between the ages of 12 and 17 years old. The results indicated that stress, ineffectiveness and parental impulsivity increase the negative effect of punitive discipline on CPV. There is no moderating effect of parental warmth/support. The gender of the aggressor is only a moderator in the case of violence toward the father, and the effect of punitive discipline is stronger in males than in females. The study draws conclusions regarding the importance of context and the mode by which parents discipline their children, aspects that can aggravate the adverse effects of physical and psychological punishment on CPV. It is necessary for interventions to focus not only on promoting positive disciplinary strategies but also on the mode in which they are administered and on contextual aspects.

## 1. Introduction

Violence of children toward their parents is a form of domestic violence that is currently internationally recognized as a social problem [1]. Child-to-parent violence (CPV) is defined as “any act by a child that is intended to cause physical, psychological or financial damage to gain power and control over a parent” [2] p. 3. More recently, other authors have noted that this type of violent behavior also aims to dominate parents [3,4]. CPV has increased dramatically in the last decade in different countries, which has led to an increase in research in this area (e.g., [5,6,7,8]). In different retrospective studies with young people aged 18–25 years, rates of verbal and psychological violence toward parents have been found to range from 72.2–97.1%, while rates of physical violence range from 4.7–15% [9,10,11]; reported rates of economic violence and control behaviors are 67% and 70%, respectively [9].

CPV has been related to various individual, family and social factors [6,12]. Given the context in which this type of violence takes place, the study of family variables takes on special importance, and, among them, exposure to family violence has received the most attention in the literature on this topic (e.g., [7,11,13,14,15,16]). Studies from the perspective of the intergenerational transmission of violence (e.g., [17,18,19]) propose that as a result of observational learning and imitation of adult models [20], children from violent homes are more likely to become violent since they internalize that aggression as an adequate way to cope with interpersonal conflicts. This exposure to violence at home can occur through the observation of violence (when children witness violence between their parents, for example) and through direct victimization (when children suffer violence from their parents). Consistent with this approach, recent studies have found that CPV is related to exposure to violence between parents (e.g., [7,11,13,21]) and to parent-to-child violence (e.g., [5,11,13,22]).

Regarding the use of violence by parents, numerous studies have shown the negative effects of aggressive or punitive discipline (PD), such as its association with problems of externalization and internalization (e.g., [23,24,25,26]). A punitive parenting style is characterized by the enforcement of rigid rules, verbal and physical hostility and high penalties for errors [27,28], including physical punishment and psychological aggression [29]. The literature indicates that CPV is related to severe disciplinary styles that employ physical and psychological punishment (e.g., [5,21,30,31,32]).

However, exposure to punitive parenting does not have the same effects on all children [33]. While for some it may be closely related to the subsequent development of violent behavior, for others, it is not significantly associated with violent behavior. The focus on risk and protective factors [33,34] has proven to be a useful theoretical framework for studying the consequences of exposure to violence from parents. This approach proposed that certain variables interact with a certain risk factor, buffering its effects (the risk factor does not affect or affects the individual to a lesser extent). Other variables can negatively affect this relationship, amplifying the negative effect of parental violence toward children. In this way, the combination of violence from parents and these variables has a more negative effect than experiencing any of these factors separately.

Following this approach, although the literature has noted the influence of PD on CPV, several important aspects of the context and the mode in which PD is used should be considered. The same disciplinary style can have very different effects depending on the context and mode of implementation [29]. Several studies have indicated the effect of various factors related to the context and mode of administration of discipline, such as parental stress, parental ineffectiveness, parental impulsivity and parental warmth/support, on CPV.

*Parental stress*

One variable related to the parental context is parental stress. Parental stress is conceptualized as a process in which parents feel overwhelmed by the responsibilities of their parental role [35,36]. Milner [37] points out that parents experiencing high levels of stress tend to evaluate situations in a less complex way, making them more impulsive when responding to their child, a response that is associated with the more frequent use of punitive practices [38]. In this way, parental stress has been related to domestic child abuse [39,40]. In addition, CPV has also been associated with family characteristics such as parental stress [41,42,43,44,45]. Del Hoyo-Bilbao et al. [31] found a significant positive relationship between parental stress and psychological aggression toward the father and the mother.

*Parental ineffectiveness*

A parenting practice is considered effective if it produces the desired result [46]. Parental ineffectiveness is also considered a variable of the context in which parental discipline is applied [29]. Research on parenting practices indicates that ineffective parenting is related to the development of behavioral problems in children [47,48], criminal behavior during adolescence [49] and problems with psychological adjustment during adulthood [50], and that behavioral problems continue due to the ineffectiveness of parental discipline [51]. Research regarding the relationship between parental ineffectiveness and CPV is quite scarce, although the data indicate that the relationship is positive. In a recent study, Del Hoyo-Bilbao et al. [31] found that ineffective parental discipline is significantly and positively related to CPV toward the mother and father. Along the same lines, in a qualitative study, the parents of adolescents recognized that their attempts to control their children’s behavior did not have the desired effects since their children did not obey them and, consequently, the parents had difficulties following through with the consequences for bad behavior that they had previously established with their children [52].

*Parental impulsivity*

As mentioned above, the same disciplinary behavior can have different effects depending on how it is implemented. For example, if a parenting practice is applied impulsively, even if it is not punitive, it is very likely to teach children to act impulsively [29]. However, studies on parental impulsivity in disciplinary behaviors are very scarce. Strauss and Mouradian [53] found that the negative effect of physical punishment on the antisocial behavior of children was stronger when mothers impulsively applied this type of disciplinary behavior. In terms of violence toward parents, Del Hoyo-Bilbao et al. [31] observed that parental impulsivity when exercising discipline is related to PD, and this in turn is related to CPV toward the father and the mother.

*Lack of parental warmth/support*

A fundamental aspect of parenting style is warmth or parental support. Warmth/support reflects parents’ general tendency to be understanding, affectionate and sensitive to the needs of the child, to express their approval and to direct positive emotions and behaviors toward the child [54]. This has been negatively associated with the internalization and externalization of problems and positively associated with social competence and psychological adjustment (e.g., [55,56,57]. Low parental warmth/support has also been related to CPV [31,52,58]. Parental warmth is fundamental to the development of CPV [10] and is a protective factor against the physical violence of children toward parents [5]. In a recent study, Cano-Lozano et al. [59] delved into the role of perceived parental warmth/support in CPV and found that parental warmth/support was negatively correlated with hostile attribution, adolescent anger and having a relationship with a deviant peer group. In turn, hostile attribution and anger in adolescents predicted CPV with reactive motivations, while involvement with a deviant peer group was associated with drug use, which in turn predicted both reactive and instrumental CPV.

Although it has been possible to verify the relationship of various aspects of the parental context and mode of implementation of parental discipline with CPV, few studies have examined the moderating effect of these variables on the relationship between PD and CPV. Parental context variables can act as amplifiers or buffers of this relationship. Specifically, the moderating role of a positive parenting context has been studied. Gámez-Guadix et al. [60] found that corporal punishment is associated with a greater likelihood of antisocial traits and behaviors regardless of whether it is used in a positive parenting context. Results along the same lines have been found specifically in relation to CPV. Corporal punishment is associated with a higher probability of psychological CPV over time, regardless of whether a positive parental context exists [30]. Beckman [61] analyzed the moderating role of family relationships (family cohesion, family conflict and interparental violence) in the relationship between physical punishment and CPV. The results indicated that family cohesion cushioned the harmful effects of parental violence on physical CPV, while family conflict exacerbated this link. Interparental violence did not have a moderating effect. Regarding parental warmth/support and its possible role in buffering the effect of PD on the psychological adjustment of the child, the results are contradictory. Some studies have found that parental warmth does not moderate the associations between punishment and children’s externalization and internalization issues (e.g., [24,30,62]. In contrast, other studies indicate that PD does not have negative consequences when used by affectionate and understanding parents [25,63,64] and that parental warmth dampens the negative influence of punishment (e.g., [65,66,67]. Finally, several studies have found that the warmth of parents can intensify the negative influence of PD. In a longitudinal study [68], anxiety increased over time in cases in which both punishment and warmth were intense, while it decreased when punishments were severe and there was minimal warmth. In this study, parental warmth did not moderate the relationship between punishment and any change in aggression. Anonas and Alampay [69] found a similar pattern with respect to verbal punishment: high maternal warmth increased the detrimental effect of verbal punishment on internalizing and externalizing behaviors. In addition, in another longitudinal study, the combination of intense punishment and intense warmth predicted behavioral problems [70].

Finally, regarding the moderating role of gender in the relationship between PD and CPV, some studies found that gender does not moderate the association between PD and antisocial behavior problems in adolescents [60] or the association between PD and CPV [30], which suggests that PD increases the probability of CPV, regardless of the gender of the adolescent.

In conclusion, the scientific literature highlights the importance of context and the mode in which discipline is administered to alter the relationship between PD and CPV. However, some variables of great interest, such as stress, impulsivity and parental ineffectiveness, have not been studied. For other variables, such as parental warmth/support, the results are contradictory; therefore, studies that provide clarification are necessary.

For this reason, the current study aimed to examine the moderating role of some variables of the context and the mode of implementation of parental discipline on CPV. Concretely, the following objectives were proposed:

(1) To analyze the relationship between PD and CPV toward the father and mother.
(2) To examine the moderating role of the parental context (parental stress and ineffectiveness) and the mode of implementation of parental discipline (impulsivity and parental warmth/support), as well as the gender of the aggressor, in the relationship between PD and CPV toward the father and mother.

Consequently, the following hypotheses were established:

**Hypotheses** **1** **(H1).** *PD will be significantly and positively associated with CPV toward the father and mother (e.g., [21,31,32])*.

**Hypotheses** **2** **(H2).** *Given the differential effect of parental context on disciplinary practices [29], the effect of PD on CPV will intensify in the presence of both parental stress and ineffectiveness.*

**Hypotheses** **3** **(H3).** *Because the mode in which discipline is implemented influences the effects of disciplinary practices [29], both impulsivity and parental warmth/support will increase the effect of PD on CPV.*

**Hypotheses** **4** **(H4).** *PD will be significantly and positively associated with CPV, regardless of the gender of the aggressor [30,60].*

## 2. Materials and Methods

### 2.1. Participants

The sample consisted of 1543 university students between 18 and 25 years of age (50.2% males, M_age_ = 19.9 years, SD = 1.9). The majority (73.5%) were from the University of Jaén, 25.1% were from the University of Oviedo and the rest (1.4%) were from other Spanish universities. The students represented a total of 36 different degree programs.

### 2.2. Instruments

Sociodemographic variables. A brief ad hoc questionnaire was used to gather information about the students’ ages, genders, degree programs and nationalities.

For the following variables, the participants reported retrospectively on their experiences between the ages of 12 and 17 years.

CPV was measured with the Child-to-Parent Violence Questionnaire (CPV-Q), youth version [9]. The CPV-Q consists of 19 parallel items (applied to the father and the mother; α father = 0.85; α mother = 0.85) that demonstrate different CPV behaviors: psychological, physical, financial and control/domain. The participants indicated the frequency with which they exhibited these behaviors when they were 12 to 17 years old. Each of the items is answered using a 5-point scale: 0 (never = never occurred), 1 (rarely = occurred once), 2 (sometimes = occurred two or three times), 3 (often = occurred four or five times) and 4 (very often = occurred six times or more). The instrument also includes eight items regarding the reasons for CPV, which are measured using a 4-point scale (0 = never, 1 = sometimes, 2 = almost always and 3 = always). For this study, the first part of the instrument was used.

PD was measured using the corporal punishment and psychological aggression subscales of the Dimensions of Discipline Inventory (DDI-C; [29]; Spanish version [60]). The subscales have 8 parallel items each (α father = 0.75; α mother = 0.78) that are answered with 5 response categories ranging from 0 (never) to 4 (almost always or always).

Parental stress and the ineffectiveness of parental discipline refer to the parental discipline context and were measured with 2 (α father = 0.44; α mother = 0.46) and 3 (α father = 0.68; α mother = 0.70) parallel items, respectively, from Section D of the DDI-C [29,60] using 5 response categories ranging from 0 (never) to 4 (almost always or always).

Impulsivity and parental warmth/support when applying discipline refer to the mode parents implement disciplinary measures. They were measured with 2 (α father = 0.67; α mother = 0.67) and 3 (α father = 0.68; α mother = 0.72) parallel items, respectively, from Section D of the DDI-C [29,60] using 5 response categories ranging from 0 (never) to 4 (almost always or always).

### 2.3. Design and Procedure

Descriptive research was conducted using cross-sectional surveys [71]. First, permission was obtained from the Ethics Committee of the University of Jaén (Spain) to conduct this study (Ref. CEIH 270215-1). Nonprobabilistic sampling was used to recruit the participants. The sample was obtained through incidental sampling at different Spanish universities. We attempted to match the sample in terms of gender and to include the maximum number of university degree programs. Selection was restricted by nationality (Spanish) and age range (18–25 years). Before administering the questionnaires, the students received written information about the research and signed a consent document. Paper-and-pencil questionnaires were administered in a group manner in the students’ classrooms. Participation was anonymous and voluntary. The evaluation was carried out by 3 members of the research team who were specifically trained in this protocol.

### 2.4. Data Analysis

The level of statistical significance established for all analyses was 0.05. Before the analytical treatment, the missing values were imputed using the *MICE* package in r (R Core Team (2020). R: A language and environment for statistical computing. R Foundation for Statistical Computing, Vienna, Austria) [72]. Imputation was performed for each of the subscales in isolation. No data were imputed when the participants provided less than 10% of the data for the scale; such data were considered missing values for those participants for that scale. The factors measured through the scales were averaged for each participant, and these values were considered our variables under analysis (CPV toward father: CPV-F; CPV toward mother: CPV-M; punitive discipline: PD; parental stress: PS; ineffective parental discipline: IPD; parental impulsiveness: PI; warmth/support: WS). The direct relationships between PD and the variables CPV-F and CPV-M were analyzed. Finally, the moderating roles of the variables PS, IPD, PI, WS and gender on the relationship between PD and CPV-F and CPV-M were analyzed. Regression and moderation analyses were performed with *jamovi* [73]. To analyze moderating effects, the *jamovi medmod* package was used.

## 3. Results

A linear regression analysis was used to assess the direct relationship between PD and the CPV-F and CPV-M factors. The results showed a significant and positive relationship for both factors, *β* = 0.48, *t*(1490) = 21.24, *p* < 0.001 for CPV-F and *β* = 0.49, *t*(1478) = 21.78, *p* < 0.001 for CPV-M.

Table 1 summarizes the results obtained in the moderation analysis. The rows highlighted in gray show the moderation effect for each variable. This analysis independently evaluated the influence that each of the proposed moderating variables had on the relationship between the predictor variable, PD, and the dependent variables, CPV-F and CPV-M. For CPV-F, only the WS variable did not significantly moderate the relationship. The rest of the variables showed a significant positive moderating effect on the relationship between PD and CPV-P. For CPV-M, the PS and IPD variables moderated the relationship, while PI, WS and gender did not.

**Figure 1:**
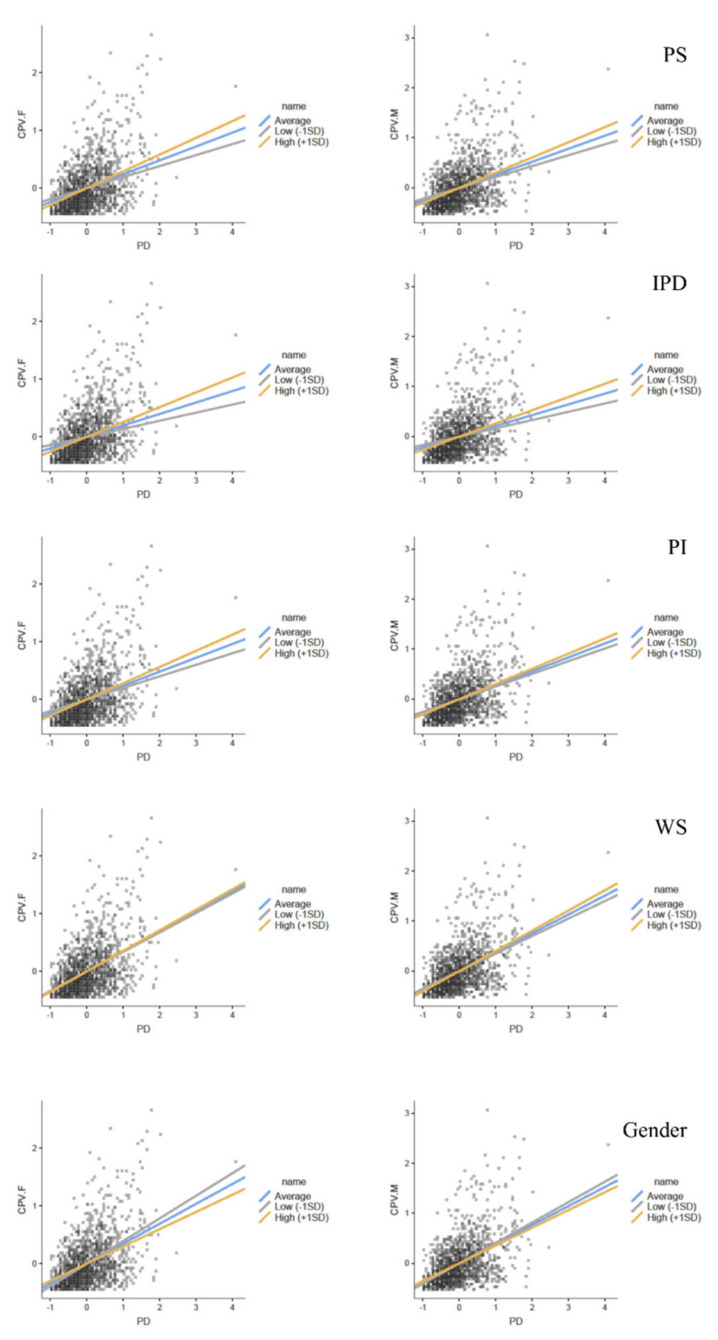
Simple slope plot of the moderating variables for CPV-F and CPV-M. Note. CPV-F: Child-to-parent violence toward father; CPV-M: child-to-parent violence toward mother; PS: parental stress; IPD: ineffective parental discipline; PI: parental impulsiveness; WS: warmth/support; SD: standard deviation. The regression lines in blue, gray and yellow show the different levels of moderation.

To determine how the moderating variables influence the relationship between the predictor variable and the dependent variable, a simple slope analysis was performed for each moderator. This analysis evaluated the effect that the predictor variable (PD) has on the dependent variables (CPV-F and CPV-M) at different levels of the moderating variable to determine the extent to which high (+1 standard deviation above the mean) and low (−1 standard deviation below the mean) levels of the moderating variable can moderate the relationship between the dependent and independent variables. Figure 1 represents the simple slope analysis for each of the moderating variables for both CPV-F and CPV-M. Through the different figures, in addition to the information on the results provided in the table, we can see not only the distribution of the original data but also the magnitude, trend and direction of the different modulation effects on the target variables, as well as the variation in modulation across the different levels of the modulating variable. In Table 1, the results of the simple slope analysis for the high and low levels are shown within the nested rows for each moderator. At all levels of analysis, even when the moderating effect was not significant, the relationships between PD and the variables CPV-F and CPV-M were significant.

## 4. Discussion

The present study examines the moderating role of the context and the mode of implementation of parental discipline on CPV. Specifically, the first objective was to analyze the relationship between PD and CPV toward the father and mother. The second objective was to examine the moderating role of parental context (parental stress and ineffectiveness) and mode of implementation of parental discipline (parental impulsivity and warmth/support), as well as the gender of the aggressor, in the relationship between PD and CPV toward the father and mother.

In Hypothesis 1, it was proposed that PD would be significantly and positively associated with CPV toward the father and mother. The results confirmed this hypothesis in both cases and corroborate the findings of several studies that indicate that this type of violence is related to severe disciplinary styles that employ physical and psychological punishment (e.g., [5,21,30,32]). The results are consistent with the hypothesis of the bidirectionality of family violence, which states that parents who are abusive toward their children, either verbally or physically, are more likely to experience CPV [74]. The results also generally agree with the extensive literature that demonstrates the negative impact of PD strategies and indicates that growing up in a violent family context is associated with subsequent aggressive behavior [75].

Hypothesis 2 predicted that the effect of PD on CPV would be more intense in the presence of both stress and parental ineffectiveness. The results confirmed this hypothesis for CPV toward both the father and the mother. Specifically, parental stress showed a moderating effect on the relationship between PD and CPV, intensifying the negative effects of PD on CPV. The relationship between parental stress and CPV has been confirmed in several studies [31,41,42,43,44,45], and the results of the present study indicate that the negative influence of PD on CPV is aggravated in the context of parental stress. In addition, it was observed that parental ineffectiveness played a moderating role in the relationship between PD and CPV, intensifying this relationship. This observation is consistent with the limited research on CPV to date, which indicates that parental ineffectiveness is associated with this type of violence [31]. However, our findings go further. Considering that, as previously indicated, parental stress and parental ineffectiveness are part of the context in which discipline is applied [29], our results indicate that in a context of parental stress or a context in which discipline is applied ineffectively (that is, parental disciplinary practices do not have the desired effect), the negative effect of PD on CPV is intensified, generating more violent behaviors toward parents. It has been noted that increased parental stress can lead to higher levels of impulsivity [37], which in turn are associated with a more frequent use of physical punishment [38]. Parental ineffectiveness could be a consequence of a history of inconsistency in establishing and enforcing rules since inconsistency in applying rules can undermine the effectiveness of any disciplinary practice [29]. To this end, in a qualitative study, parents who were victims of CPV recognized that when their children were younger, rules had been established in the home that were sometimes unclear (in cases of disagreement between the father and mother, for example) and that they had allowed their children to disobey them to avoid conflicts. They even recognized difficulties with applying the consequences for breaking the rules, such as administering physical punishment too late [76]. In such contexts, children learn that there are no serious consequences for their misbehavior since consequences are rarely applied [52], and they ignore their parents’ disciplinary efforts.

Hypothesis 3 predicted that both impulsivity and parental warmth/support would increase the effect of PD on CPV. This hypothesis was partially confirmed. Specifically, in the case of CPV toward the father, the results indicated that parental impulsivity effectively increases the intensity of the relationship between PD and CPV, although this effect was not observed in the case of CPV toward the mother. Although the relationship between parental impulsivity, PD and CPV has been previously verified [31], our study delves into the relationships among these variables. The mode in which discipline is implemented influences the effects of disciplinary practices. Previous research has found that the negative effect of physical punishment on antisocial behavior in children was stronger when punishment was applied impulsively (e.g., [53]). Our data are similar, indicating that when parents apply discipline impulsively (for example, without reflection, without adequately assessing the child’s behavior, etc.), the negative effects of PD are accentuated and generate more CPV behaviors toward the father. In addition, if a parenting practice is applied impulsively, it is very likely to teach impulsivity [29]. As a result, children learn to act impulsively, which in turn is associated with violent behaviors toward their parents [31] and, together with the learned violence derived from PD (physical and psychological punishment), further contributes to the development of CPV behaviors. The fact that impulsivity does not have a moderating role in violence toward the mother could indicate that the underlying relationships stem from mechanisms or pathways different from those involved in violence toward the father, an aspect that should be studied in greater depth.

In relation to parental warmth/support, this does not affect the intensity of the relationship between PD and CPV. The results show that PD plays a negative role in violent behavior by children toward their parents, even in the context of parental warmth/support. Several studies have found that parental warmth does not moderate the associations between punishment and externalizing or internalizing problems of the child (for example, [24,30,62]). This finding is important because it provides empirical evidence that contradicts the idea that PD has no negative consequences when used by affectionate and understanding parents [63,64]. It also does not coincide with studies suggesting that high parental warmth can increase the harmful effect of punishment [68,69,70]. It is possible that other variables, either contextual to the family or child-related, are involved in these relationships and should be taken into account in future studies to clarify this issue. Along these lines, Zubizarreta et al. [77] found that the warmth of parents can exaggerate the negative effect of a punitive parenting style when warmth is combined with certain child-related characteristics, such as temperament.

Finally, Hypothesis 4 stated that PD would be significantly and positively associated with CPV, regardless of the gender of the aggressor. This hypothesis was confirmed only for CPV toward the mother. The effect of PD on CPV toward the father is more intense among males than among females, while for CPV toward the mother, the gender of the aggressor does not moderate the relationship. The few studies that have been conducted found that gender does not moderate the association between PD and antisocial behaviors in adolescents [60], and neither does the association with CPV [30]. However, the latter study did not differentiate between violence toward the father and violence toward the mother, which may explain the differences between their results and ours.

### Limitations and Future Lines of Research

This study has some limitations that must be considered when interpreting the results. First, the data are cross-sectional, and, therefore, causal inferences cannot be made. Longitudinal studies on this topic are needed to confirm the temporal sequencing of events and to evaluate the trajectories of CPV. Second, since all the data are based on self-reports of young people, it is necessary to expand these data to include alternative sources, such as data obtained from parents. Third, information was collected retrospectively, which could lead to errors in recall. In this sense, Hardt and Rutter [78] conducted an empirical review of the information provided by participants about events that occurred during their childhood and found that the information they relayed about situations that had occurred years ago was valid. Finally, the type of sample (university students from a specific geographical and cultural environment), the sampling method used (nonprobabilistic) and the low internal consistency of some scales limit the generalization of the results to other environments, and it is necessary to replicate these results in other populations.

## 5. Conclusions

This study examines the relationships between PD and CPV in young people. There is abundant literature on the subject that highlights the negative effect of severe or punitive disciplinary practices, such as physical and psychological punishment, on the development of violent behavior toward parents. The results of this study indicate that the relationship between the two variables is moderated by other variables that intensify this relationship, such as parental stress, parental ineffectiveness and parental impulsivity. These variables, which are related to both the context in which the discipline is applied and the way in which it is implemented, can cause disciplinary practices to have variable effects, and these aspects are sometimes even more relevant than the specific disciplinary behaviors [29]. The context in which discipline is implemented (for example, if parents are stressed or if there is ineffective supervision) and the way in which it is implemented (for example, if it is applied impulsively) are extremely important aspects of discipline. They can aggravate the adverse effects of some disciplinary behaviors, such as the use of physical and psychological punishment. Furthermore, studies of CPV with samples of adults are scarce and can provide valuable information on the research topic.

Therefore, these results have various implications. First, at the research level, it is important to know not only the effects of disciplinary behaviors but also the way in which they are administered and the relevant contextual aspects of the disciplinary situation. The identification of such factors that can buffer or amplify the effects of disciplinary practices will provide a deeper understanding of the family processes that operate as CPV develops and continues. Although abusive relationships between parents and children tend to coexist within a dysfunctional family environment [68], substantial variations are expected according to the way in which parents implement discipline. Second, and related to the previous item, a better understanding of these family processes is essential for designing effective interventions in cases of family violence. On the one hand, as a preventive approach, families should be informed and sensitized about the negative consequences of PD. Families should receive training on adaptive conflict resolution strategies and the use of positive teaching practices that do not involve corporal punishment. Strategies based on supervision and control, such as positive reinforcement of appropriate behavior, restorative behavior or deprivation of privileges, can help prevent CPV. In addition, it is necessary for interventions to focus not only on promoting positive disciplinary strategies but also on the way in which discipline is administered and the relevant contextual aspects of the disciplinary situation to help families develop skills for the adaptive and effective application of disciplinary measures.

## Figures and Tables

**Table 1:** Summary of the results of the moderation analysis.

					95% CI			
VD	Moderator	Simple Slope	Estimate	SE	Lower	Upper	*Z*	*p*
CPV-F	PS		0.06	0.02	0.03	0.09	3.68	< 0.001
		Low (−1 SD)	0.19	0.03	0.14	0.24	7.41	< 0.001
		High (+1 SD)	0.29	0.02	0.26	0.32	16.71	< 0.001
	IPD		0.07	0.02	0.04	0.10	4.92	< 0.001
		Low (−1 SD)	0.14	0.02	0.09	0.19	6.00	< 0.001
		High (+1 SD)	0.26	0.02	0.23	0.29	15.75	< 0.001
	PI		0.04	0.01	0.01	0.07	2.99	0.003
		Low (−1 SD)	0.20	0.03	0.15	0.25	7.35	< 0.001
		High (+1 SD)	0.28	0.02	0.25	0.31	16.75	< 0.001
	WS		0.01	0.02	−0.02	0.04	0.62	0.534
		Low (−1 SD)	0.34	0.02	0.30	0.38	17.21	< 0.001
		High (+1 SD)	0.36	0.02	0.31	0.40	14.46	< 0.001
	Gender		−0.09	0.03	−0.16	−0.03	−2.87	0.004
		Male	0.39	0.02	0.35	0.44	17.39	< 0.001
		Female	0.30	0.02	0.25	0.34	12.70	< 0.001
CPV-M	PS		0.05	0.02	0.02	0.09	2.98	0.003
		Low (−1 SD)	0.22	0.03	0.16	0.27	7.93	< 0.001
		High (+1 SD)	0.30	0.02	0.27	0.34	16.48	< 0.001
	IPD		0.06	0.02	0.03	0.09	3.91	< 0.001
		Low (−1 SD)	0.17	0.02	0.12	0.21	6.74	< 0.001
		High (+1 SD)	0.26	0.02	0.23	0.30	15.36	< 0.001
	PI		0.03	0.01	−0.00	0.06	1.74	0.081
		Low (−1 SD)	0.25	0.03	0.20	0.31	8.69	< 0.001
		High (+1 SD)	0.30	0.02	0.27	0.34	16.97	< 0.001
	WS		0.01	0.02	−0.02	0.04	0.62	0.534
		Low (−1 SD)	0.34	0.02	0.30	0.38	17.21	< 0.001
		High (+1 SD)	0.36	0.02	0.31	0.40	14.46	< 0.001
	Gender		−0.05	0.03	−0.12	0.02	−1.51	0.131
		Male	0.41	0.02	0.36	0.45	16.91	< 0.001
		Female	0.35	0.03	0.31	0.40	14.13	< 0.001

Note. CPV-F: Child-to-parent violence toward father; CPV-M: child-to-parent violence toward mother; PS: parental stress; IPD: ineffective parental discipline; PI: parental impulsiveness; WS: warmth/support; SD: standard deviation. The rows shaded gray show the moderation effect for each variable.

## Data Availability

The dataset generated and analysed during the current study are not publicly available due to confidentiality reasons, but are available from the corresponding author on reasonable request.
